# Surgery After Surgery for Vestibular Schwannoma: A Case Series

**DOI:** 10.3389/fonc.2020.588260

**Published:** 2020-12-18

**Authors:** Łukasz Przepiórka, Przemysław Kunert, Wiktoria Rutkowska, Tomasz Dziedzic, Andrzej Marchel

**Affiliations:** Department of Neurosurgery, Medical University of Warsaw, Warsaw, Poland

**Keywords:** vestibular schwannoma, surgery, revision, facial nerve, gross total resection

## Abstract

**Objective:**

We retrospectively evaluated the oncological and functional effectiveness of revision surgery for recurrent or remnant vestibular schwannoma (rVS).

**Methods:**

We included 29 consecutive patients with unilateral hearing loss (16 women; mean age: 42.2 years) that underwent surgery for rVS. Previous surgeries included gross total resections (GTRs, n=11) or subtotal resections (n=18); mean times to recurrence were 9.45 and 4.15 years, respectively. House–Brackmann (HB) grading of facial nerve (FN) weakness (grades II-IV) indicated that 22 (75.9%) patients had deep, long-lasting FN paresis (HB grades: IV-VI). The mean recurrent tumor size was 23.3 mm (range: 6 to 51). Seven patients had neurofibromatosis type 2.

**Results:**

All patients received revision GTRs. Fourteen small- to medium-sized tumors located at the bottom of the internal acoustic canal required the translabyrinthine approach (TLA); 12 large and small tumors, predominantly in the cerebellopontine angle, required the retrosigmoid approach (RSA); and 2 required both TLA and RSA. One tumor that progressed to the petrous apex required the middle fossa approach. Fifteen patients underwent facial neurorrhaphy. Of these, 11 received hemihypoglossal–facial neurorrhaphies (HHFNs); nine with simultaneous revision surgery. In follow-up, 10 patients (34.48%) experienced persistent deep FN paresis (HB grades IV-VI). After HHFN, all patients improved from HB grade VI to III (n=10) or IV (n=1). No tumors recurred during follow-up (mean, 3.46 years).

**Conclusions:**

Aggressive microsurgical rVS treatment combined with FN reconstruction provided durable oncological and neurological effects. Surgery was a reasonable alternative to radiosurgery, particularly in facial neurorrhaphy, where it provided a one-step treatment.

## Introduction

Vestibular schwannoma (VS) surgery has changed beyond recognition since the early 20^th^ century, when it was associated with mortality rates as high as 86% (1). The subsequent drop to 15.4% mortality, due to contributions from Harvey Cushing, was considered a milestone in VS surgery (2). In contrast, contemporary skull base surgery includes gross total resections (GTRs), but also aims for a good functional outcome, mostly by preserving facial nerve (FN) and other cranial nerve functions (3–6) in addition to reducing the mortality rate (7, 8).

Treatment options for VS include observation, surgery, *via* a retrosigmoid approach (RSA) (9–11), middle fossa approach (12), or translabyrinthine approach (TLA) (13), and stereotactic radiosurgery (SRS) (14–17). Additionally, some physicians prefer a combination of treatment methods, including an intentional partial resection, followed by SRS (18–20). Unfortunately, some patients experience tumor progression after a subtotal resection or recurrence after a GTR. Recurrences are uncommon; rates range from 0.3% to 9.2% (21). However, regrowth of a residual tumor occurs in up to 44% of cases (22).

Tumor recurrence or progression management remains controversial. In our department, we follow the VS management principles described by Samii, namely a GTR with the preservation of facial and cochlear nerve functions (23). A second microsurgery is much more difficult to perform than an initial GTR, due to adhesions and scarring, and it is even more difficult after a radiosurgery (24). In this retrospective case study, we described a series of patients that underwent revision surgery for VS and, in some cases, they received different methods of FN reanimation (25–27). In this study, we aimed to evaluate the oncological and functional effectiveness of revision surgery for recurrent and residual VS (together referred to as rVS).

## Materials and Methods

This study was designed as a retrospective, single-center, consecutive case series, undertaken in an academic setting. All participants met the following criteria:

- Previous history of surgery for a VS, with or without radiation therapy;
- Histopathologic diagnosis of a VS, during the first and second surgeries;
- Regrowth, diagnosed in radiological imaging, or a residual tumor in patients with FN palsy;
- Revision surgery required for VS.

No specific exclusion criteria were applied. Patients underwent reoperations from 2002 to 2018. Data were collected between 2018 and 2019. We analyzed 29 patients (16 women, 13 men; mean age: 42.2 years) that underwent surgery for rVS. The mean time to revision surgery was 6.16 years (**Table 1**).

**Table 1:** Surgical and postoperative characteristics of 29 patients treated for vestibular schwannoma.

Characteristic	Data	
**Mean age, years (range; median)**	42.2 (22−68; 46)	
**Sex**		
**Males:**	13 (44.8)	
**Females:**	16 (55.2)	
**Initial surgery**		
**Our Department:**	11 (37.9)	
**Other centers:**	18 (62.1)	
**Initial surgery, extent of resection**		
**GTR:**	11 (37.9)	
**Non – GTR**	16 (55.2)	
**N/A:**	2 (6.9)	
**Mean time to revision surgery, years (range)**	6.16 (0.5−19)	
**Facial neurorrhaphy:**	15 (51.7)	
**No facial neurorrhaphy:**	14 (48.3)	
**Mean time to facial neurorrhaphy, years (range)**	1.5 (0.5−4)	
**History of radiosurgery:**	4 patients (13.8)*	
**Neurofibromatosis type 2**	7 patients (24.1)	
**Side of the tumor**		
**Right:**	16 (55.1)	
**Left:**	13 (44.9)	
**Location of the recurrence**		
**IAC:**	9	
**IAC and CPA:**	18	
**CPA:**	1	
**Petrous apex:**	1	
**Mean maximal tumor size before revision surgery, mm (range)**	23.3 (6 −51)	
**Revision surgery – approach**		
**TLA:**	14 (48.3)	
**RSA:**	12 (41.4)	
**RSA and TLA:**	2 (6.9)	
**MFA:**	1 (3.4)	
**Mean follow-up after revision surgery, years (range; median)**	3.46 (0.25−11; 2)	
**Modified Rankin Scale:**	Before revision surgery	After revision surgery, at follow-up
**grade 0**	0 (0)	0 (0)
**grade 1**	0 (0)	17 (58.7)
**grade 2**	0 (0)	9 (31)
**grade 3**	28 (96.6)	2 (6.9)
**grade 4**	0 (0)	0 (0)
**grade 5**	1 (3.4)	1 (3.4)

Values are n (%) or the mean (range), as indicated. *Twice for one patient.

CPA, cerebellopontine angle; IAC, internal acoustic canal; GTR, gross total resection; MFA, middle fossa approach; N/A, not available; RSA, retrosigmoid approach; TLA, translabyrinthine approach.

Eleven patients (37.9%) had undergone previous GTR surgeries in our department with the retrosigmoid approach (RSA). In this group, the mean time to revision surgery was 9.45 years (range 4 to 19). Eighteen other patients (62.1%) were referred from other centers, due to tumor progression after subtotal resections (all RSA); among these, 4 patients had undergone additional radiosurgery. The mean time to revision surgery was 4.15 years (range 0.5 to 16). Over one third (37.9%; 11/29) of the initial surgeries were GTRs; 55.2% (16/29) were subtotal resections; and 2 were unknown, due to the lack of available data.

All patients presented with unilateral hearing loss (American Academy of Otolaryngology-Head and Neck Surgery class D). Patients had different grades of FN weakness (House–Brackmann[HB] grades: II–VI). Most patients (75.8%) experienced deep FN paresis (HB grades: IV–VI; **Tables 1**, **2**). Recurrent tumor sizes ranged from 6 to 51 mm (mean: 23.3 mm). Seven patients (24.1%) had neurofibromatosis type 2 (NF2). In those cases, surgery notes were reviewed to ensure that the tumor did not originate from a location other than the eighth cranial nerve.

**Table 2:** House-Brackmann grades of vestibular schwannoma, before and after revision surgery.

House-Brackmann grades	Before revision surgery	Long-term follow-up after revision surgery*
**I**	0 (0)	1 (3.4)
**II**	1(3.4)	3 (10.3)
**III**	6 (20.7)	15 (51.8)
**I – III**	7 (24.1)	19 (65.6)
**IV**	2 (6.9)	4 (13.8)
**V**	0 (0)	1 (3.4)
**VI**	20 (69)	5 (17.3)
**IV – VI**	22 (75.9)	10 (34.5)
**Total**	**29 (100)**	**29 (100)**

*Surgeries were performed with or without a facial neurorrhaphy.

The following pre-intervention procedures were included specifically for this study: when possible, descriptions of previous surgeries were acquired, and current scans were performed with magnetic resonance imaging (MRI), electromyography for the FN, and a bone window computed tomography (CT) for the temporal bone. Otherwise, all patients were prepared for elective surgery in a routine fashion. Hearing was not tested, because all patients were unilaterally deaf.

## Results

The interventions included revision surgery, tumor removal, and facial neurorrhaphy (n=15). One patient had undergone a facial neurorrhaphy prior to revision surgery, and 14 patients underwent tumor resections simultaneous with facial reanimation surgeries. Most facial reanimation procedures were hemihypoglossal-facial neurorrhaphies (80%, 12/15). The classic hypoglossal-to-facial nerve neurorrhaphy technique was applied in 3 (20%, 3/15) cases.

### Surgery Details

**Figure 1:**
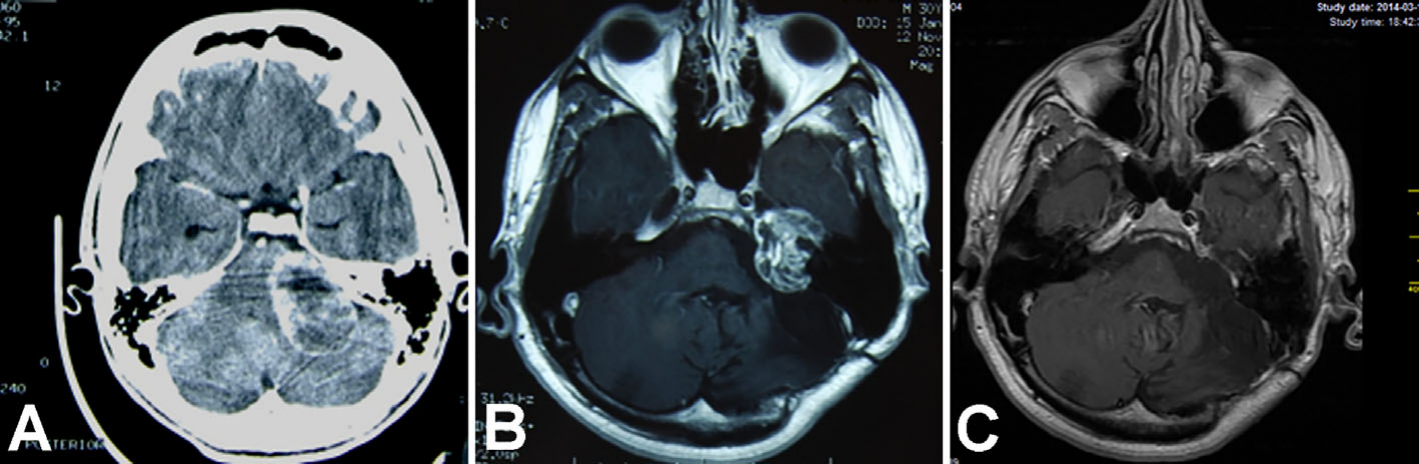
Magnetic resonance T1WI contrast enhanced axial images show treatment results in an 18-year-old man with vestibular schwannoma. **(A)** Brain scan before the initial surgery; **(B)** image after the initial surgery; **(C)** image after revision surgery *via* a middle fossa approach.

Each surgery was performed under general anesthesia, with the patient positioned on the back, with the head rotated contralaterally. The TLA was used for 14 small- to medium-sized tumors (median maximal size: 15.63 mm) that arose from the bottom of the internal acoustic canal (IAC). The RSA was used for larger tumors (median maximal size: 28.33 mm) and for smaller tumors (n=12) that were predominantly located in the CPA. In two cases, a combination of TLA and RSA was performed. The middle fossa approach was employed for one tumor that had progressed to the petrous apex (**Figure 1**).

Intraoperative neurophysiological monitoring was performed only in selected cases. Hearing was not monitored, because all patients were unilaterally deaf. FN was monitored in patients that had at least partially preserved FN function after a previous surgery.

Patients that underwent facial neurorrhaphy were instructed by a neurophysiotherapist to perform self-massaging of the facial muscles postoperatively. Then, when the first signs of muscle reinnervation appeared, patients were to perform exercises in front of the mirror. Patients were monitored postoperatively, as follows: outpatient clinic visits at ½, 1, 1.5, and 3.5 years after surgery; and MRIs at ½, 1,5, and 3.5, years after surgery.

### General and Oncological Results

There were no deaths. Postoperative complications occurred in four patients (13.8%). These included one subcutaneous hematoma, after an abdominal fat harvest (treated with a wound revision); one otorrhea (treated with a temporary lumbar drainage); one rhinorrhea (treated with duraplasty); and one otorrhea combined with chronic otitis media (treated with temporary lumbar drainage and a petrosectomy). The cerebrospinal fluid (CSF) leak rate was 10.3% (3/29).

In all cases but one, GTR was accomplished during the second surgery. The one unsuccessful GTR was performed in a patient admitted in poor general condition (Modified Rankin Scale 5), and revision of a previous partial VS resection improved his neurological deficits, but not significantly. Among the other 28 patients, no subsequent tumor recurrence was noted during amen follow-up of 3.46 years (range: 0.25–11).

### Facial Nerve Functional Results

The FN function improved during follow-up in 69.0% (20/29) of patients, and it remained unchanged in 24.1% of patients (7/29). Additionally, two patients (6.9%, 2/29) experienced worse FN function after revision surgery. Both of those patients had been initially treated in our department, 8 and 9 years prior to revision surgery. They initially had HB grades of II and IV, and after revision surgery, their HB grades were IV and VI, respectively. Neither of these patients was eligible for facial neurorrhaphy, due to HB grade IV, in the former patient, and long-lasting FN paralysis, in the latter patient. When analyzed separately, 71.4% of patients (5/7) with NF2 experienced better FN function after revision surgery, and in 28.6% (2/7) it remained unchanged.

Fifteen patients received facial neurorrhaphies. Of these, 12 had hemihypoglossal–facial neurorrhaphies (HHFNs). Of the latter, 9 underwent an HHFN simultaneously with the revision surgery. In follow-up, 10 patients (34.48%) experienced continued deep FN paresis (HB grades IV-VI). Of these, 8 (80%, 8/10) had not received FN reanimation. Of patients with NF2, 42.9% (3/7) had HHFNs: 2 simultaneously with the revision surgery and 1 afterwards. After HHFN, all patients improved from HB grade VI to HB grade III, except one patient that improved to grade IV (**Table 3**). In summary, HB grades I–III were observed in 24.1% (7/29) of patients before revision surgery and in 65.5% (19/29) during follow-up.

**Table 3:** Facial nerve function after revision surgery, according to the presence, technique, and time to facial neurorrhaphy.

Procedure	Total number of patients	House-Brackmann grading scale at follow–up					
		I	II	III	IV	V	VI
**Revision surgery:without facial neurorrhaphy**	14	1	3	2	2	1	5
		6			8		
**with facial neurorrhaphy**	15	0	0	13	2	0	0
		13			2		
**HHFN**	12	0	0	11	1	0	0
**Classic XII – VII neurorrhaphy**	3	0	0	2	1	0	0
**Facial neurorrhaphy:≤1 year after FN paralysis onset**	11	0	0	10	1	0	0
		10			1		
**>1 year after FN paralysis onset**	4	0	0	3	1	0	0
		3			1		

HHFN, hemihypoglossal-facial neurorrhaphy; FN, facial nerve.

During the last 5 years of the study (revision surgeries from2013 to 2018), 14 patients had available data. Of these, 9 received the HHFN technique, and the results were satisfactory results in each case. Due to our complex surgical strategy, 11 (78.6%) patients moved from an unsatisfactory functional grade (HB grades VI–VI) to a satisfactory functional grade (HB grades I–III, **Table 4**).

**Table 4:** Facial nerve outcomes in 14 patients treated for vestibular schwannoma during the last 5 years of the study.

House-Brackmann grades	Before revision surgeryn (%)	Long-term outcomen (%)
**I**	0 (0)	0 (0)
**II**	0 (0)	0 (0)
**III**	0 (0)	11 (78.6); 9*
**IV**	3 (21.4)	1 (7.13)
**V**	0 (0)	1 (7.13)
**VI**	11 (78.6); 9*	**1 (7.13)**

*Number of patients that underwent a hemihypoglossal–facial neurorrhaphy.

### Illustrative Case 1—Middle Fossa Revision and Classic Facial Neurorrhaphy

An 18–year old male underwent a GTR with an RSA for a sporadic VS in our department. Then, he underwent a classic hypoglossal – facial neurorrhaphy. His FN function improved to HB grade IV. Eight years later, he was diagnosed with a recurrent tumor, with a maximal size of 33 mm, which had progressed toward the petrous apex. The patient underwent revision surgery *via* a middle fossa approach, and the tumor was totally resected. The postoperative course was uneventful. No subsequent tumor regrowth was noted in a follow-up of 11 years (**Figure 1**).

### Illustrative Case 2—Retrosigmoid Revision and Hemihypoglossal-Facial Neurorrhaphy

A 54–year old female presented with FN paresis after a subtotal VS resection in another center one year earlier. The neurological examination at admission revealed deafness, right sided dysmmetry, dizziness, subjective diplopia, hypoaesthesia on the right side of the face, and paresis of the soft palate, but no difficulties in swallowing. Her follow-up MRI revealed a tumor remnant, located in the right CPA, intrameatally. She was eligible for revision surgery *via* RSA with a simultaneous HHFN.

**Figure 2:**
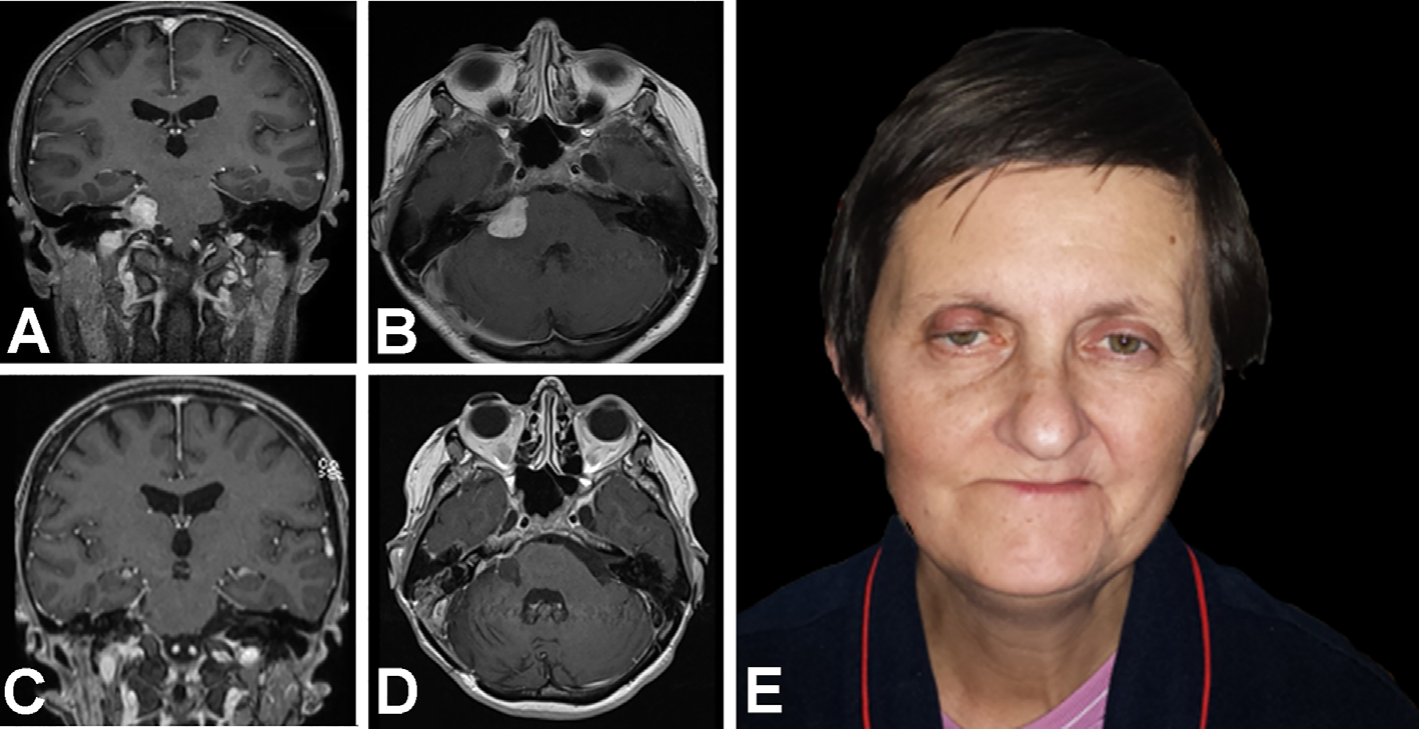
Magnetic resonance T1WI contrast enhanced images and photograph show treatment results in a 54–year old woman with facial nerve paresis after a subtotal resection of a vestibular schwannoma. At one year after the initial surgery, **(A)** coronal and **(B)** axial MR images show growth after stereotactic radiosurgery. Then, revision surgery was performed, *via* a retrosigmoid approach with facial neurorrhaphy; 11 months later, **(C)** coronal and **(D)** axial T1WI MR images show the results of a gross total resection with **(E)** satisfactory facial nerve function.

After revision surgery, the patient developed hypoglossal paresis, which subsequently resolved. Two months later, the patient developed otorrhea with chronic otitis media. A CT revealed a fistula between CPA, mastoid air cells and the external acoustic canal. The patient underwent petrosectomy. The subsequent postoperative course was uneventful; her follow-up MRI confirmed the completeness of the VS resection. In a 2–year follow–up, FN function improved to HB grade III (**Figure 2**).

### Illustrative Case 3—Translabyrinthine Revision and Hemihypoglossal-Facial Neurorrhaphy

**Figure 3:**
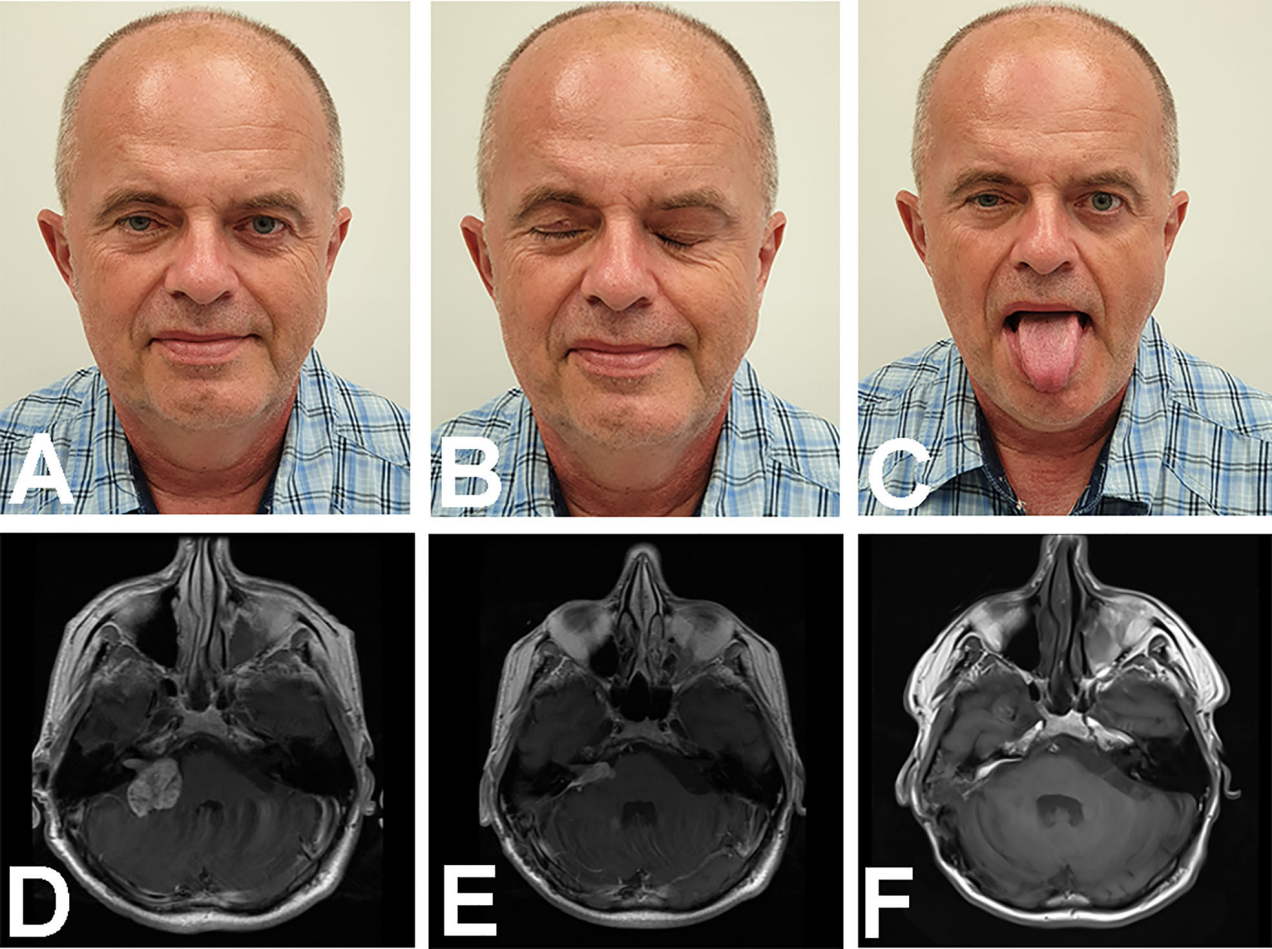
Images show treatment results in a 53–year old male with right facial nerve and abducens nerve pareses and right-sided deafness, after a partial VS removal. Photographs show the patient with the face **(A)** at rest, **(B)** with closed eyes, and **(C)** when asked to put out his tongue. The latter image is significant, because it shows no visible muscular atrophy and a straight position. **(D−F)** Magnetic resonance T1WI images with contrast enhancement show the brain **(D)** before, and **(E)** after the first surgery, which resulted in a remnant tumor. **(F)** The brain after revision surgery *via* a translabyrinthine approach.

A 53–year old male presented with right facial and abducens nerve paresis and right-sided deafness after a partial VS removal 6 months earlier in another center. A tumor remnant was located close to the porus acusticus. He was eligible for a tumor remnant resection with a TLA and simultaneous HHFN. The postoperative course was uneventful. He was discharged one week after surgery. At a 1–year follow-up, his FN function improved to HB grade III, and a postoperative MRI revealed no tumor remnant (**Figure 3**).

## Discussion

### Vestibular Schwannoma Recurrences and Remnants

The source of a VS recurrence might be a microscopic tumor remnant on the cranial nerves or in the fundus of the internal auditory canal (24). To address these possibilities, various attempts have been made to maximize the VS resection *via* a retrosigmoid or middle fossa approach; i.e., with endoscopy (28–30). However, in a previous retrospective study, Panigrahi et al. found that the Ki-67 labeling index, rather than the extent of resection, was associated with VS recurrences (31). Additionally, Freeman et al. hypothesized that a recurrence or regrowth after a GTR was more likely to reflect the biological behavior of the individual tumor, rather than the size of a tumor residue (32).

On the other hand, the number and relative contribution of revision surgeries for growing tumor remnants will probably increase, due to the current popularity of less invasive approaches, like non-total “functional” resections, with or without radiosurgery (20, 33). Therefore, complex solutions should be developed for the most demanding resections.

### Management Options

There are three therapeutic options for treating a VS, including observation, revision surgery, and radiosurgery. According to Tomita et al., a small remnant VS after microsurgery could be managed conservatively without additional treatment (34). Similarly, Troude et al. preferred a “wait and scan policy” over a postoperative SRS (35). Although the “wait and scan” policy is advantageously non-invasive, it was previously observed that, in certain patients with VS, the quality of life was mostly improved after microsurgery. However, one potential explanation for that result might be that the patient was not told that he/she had an intracranial tumor; this knowledge appeared to cause an intolerable burden, regardless of the tumor size, even though tumors often remained stable in observation groups (36, 37). Revision surgery is the only method that actually removes a tumor, but the trade-off is the high risk of postoperative complications, compared to other alternatives. Stereotactic radiosurgery is a less invasive alternative to microsurgery, but it does not actually eliminate the tumor. Moreover, large or giant tumors usually cannot be treated with SRS; therefore, they require microsurgical resection (38, 39). Furthermore, it should not be forgotten that, although rare and controversial, a VS can be malignantly transformed after a SRS, and radiation can induce a VS (40–44).

### Aim of the Revision Surgery

The aim of the second surgery should be an oncological cure and preserving FN function. Alternatively, in cases with FN paralysis, neurorrhaphy techniques should be carried out alongside the tumor resection, when the time period after paralysis onset allows. In contrast to the initial VS treatment, a second surgery does not usually aim to preserve hearing, because the patients are unilaterally deaf.

### Complications

Several previous investigations of microsurgery for rVS showed satisfactory results and low complication rates (45). The rate of postoperative complications in our series was 10.3%, and the CSF leak rate was 13.8%. Three patients required different surgical revisions without intradural CPA inspections. Perry et al. described two cases in a 6-patient series where a CSF leak (33%) was successfully treated with a lumbar drain and surgical revision. Their summary of a previous series indicated a 4% CSF leak rate (45). Apart from that study, no other study reported major complications (24, 32, 45, 46).

### Approach Selection

When considering the rVS treatment approach, the surgeon should bear in mind both the location of the tumor and its size. Typically, the approach should be different than that previously used to avoid scar tissue, which can adhere tightly to the cranial nerves, brainstem, or cerebellum (32). In our experience, the TLA was most suitable for resecting minor remnants, when the primary aim was an HHFN, because, after skeletonizing the FN in the Fallopian canal, limited expansion of the bony drilling was required to access the tumor. On the other hand, for major tumor progressions toward the brain stem or petrous apex, the RSA and middle fossa approach are also reasonable choices.

### Oncological Results

In a literature review of microsurgical treatments for rVS, Perry et al. reported no recurrences after a mean follow-up of 55 months. That finding was consistent with our present findings with revision surgery; we found no recurrences after a mean follow-up of 41.5 months. In comparison, Huang et al. recently reported a 94% tumor control rate with SRS for rVS after a median clinical follow–up of 74 months (47).

### Functional Results

In our series, the rate of deep FN deficits (HB grades IV-VI) was reduced from 75.9% to 34.5% in the long-term follow-up (p <0.05, Fisher’s exact test, **Table 2**). Considering only the results from the last 5 years of our study, 82% (9/11) of patients with complete FN paralysis recovered satisfactory function. According to Samii et al., the main predictor of FN outcome in a second VS surgery was the level of facial function before revision surgery (24). Postsurgical results can be further improved by combining revision surgery with facial neurorrhaphy. In our series, after an HHFN, all patients improved from HB grade VI to HB grade III, except one, who improved to grade IV.

Fourteen of our patients did not undergo facial neurorrhaphy, partly because the timeframe was exceeded for performing this procedure (n=7). Prolonged times between the FN paralysis onset and neurorrhaphy was correlated with worse FN outcomes (27). The remaining 7 patients had initial HB grades of II to IV, which excluded facial neurorrhaphy during revision surgery. In follow–up, the HB grades of this subgroup improved, except in 3 patients: the HB grade remained unchanged in 1 patient, and it worsened in 2 patients. For patients with unsatisfactory long–term FN function, plastic surgery was advised.

A substantial number of our patients had unsatisfactory FN function, particularly during the early part of the study period. The two main explanations were that some patients did not consent to receive face reanimation procedures and some surgeons underestimated the significance of postoperative FN weakness. Nineteen patients presented with HB grade VI after the first surgery; of these, 13 (68.4%) were treated initially in centers with relatively low surgical volumes. In other words, as much as 72.2% (13/18) of patients treated in other centers had HB grade VI after surgery. This finding suggested that patients with VS should be treated preferentially in experienced, large volume centers. High-volume departments have shorter lengths of stay, lower complication rates, and reduced hospital-related costs (48, 49). Patients treated initially in our department, apart from experiencing better FN outcomes, had longer mean times to revision surgery (9.45 years) compared to other centers (4.15 years). Similarly, Sanna et al. concluded that patients with VS should be treated by highly specialized centers, which limit subtotal resection to only few selected cases without compromising postoperative morbidity (50).

### Our Strategy for Recurrent and Residual VS

We performed GTRs during revision surgery in 97%of patients, and we observed FN functional improvements in 69% of patients. Based on these results, we suggest that revision surgery should be considered when: (1) a patient requires facial neurorrhaphy and has a stable residual tumor; or (2) the tumor has regrown (here, the HHFN should be performed, when necessary). Moreover, we prefer neurorrhaphy techniques, rather than plastic surgery, up to the end of the 3^rd^ year after paresis onset (27). Additionally, it is important to provide a multidisciplinary, complex approach, from the diagnosis to late follow-up, with close surveillance of postoperative FN function.

### Study Limitations

This study was limited by its retrospective, single–center design. Additionally, patients with and without NF2 were analyzed together, despite differences in the management of these two groups.

## Conclusions

This study demonstrated that aggressive microsurgical rVS treatment, together with modern FN reconstruction techniques provided an acceptable risk profile, yielded durable oncological effects, and could restore satisfactory FN function. We found that surgery was a reasonable alternative to SRS, particularly in patients that required facial neurorrhaphy, because it offered a one-step treatment.

## Data Availability Statement

The original contributions presented in the study are included in the article/**Supplementary Material**. Further inquiries can be directed to the corresponding author.

## Ethics Statement

The studies involving human participants were reviewed and approved by The Bioethics Committee of the Medical University of Warsaw. The patients/participants provided their written informed consent to participate in this study. Written informed consent was obtained from the individual(s) for the publication of any potentially identifiable images or data included in this article.

## Author Contributions

PK designed the project and critically reviewed it. ŁP prepared the manuscript, collected and reviewed data. WR and TD collected case data. AM did final approval of the version to be published. All authors have participated sufficiently in the conception and design of this work or the analysis and interpretation of the data, as well as the writing of the manuscript, to take public responsibility for it. All authors contributed to the article and approved the submitted version.

## Conflict of Interest

The authors declare that the research was conducted in the absence of any commercial or financial relationships that could be construed as a potential conflict of interest.

## Abbreviations

CPA, cerebellopontine angle; CSF, cerebrospinal fluid; CT, computed tomography; FN, facial nerve; GTR, gross total resection; HB, House–Brackmann; HHFN, hemihypoglossal-facial neurorrhaphy; IAC, internal acoustic canal; MFA, middle fossa approach; MRI, magnetic resonance imaging; NF2, neurofibromatosis type 2; RSA, retrosigmoid approach; rVS, recurrent and residual vestibular schwannoma; SRS, stereotactic radiosurgery; TLA, translabyrinthine approach; VS, vestibular schwannoma.
